# Flexibility Correlation between Active Site Regions Is Conserved across Four AmpC β-Lactamase Enzymes

**DOI:** 10.1371/journal.pone.0125832

**Published:** 2015-05-27

**Authors:** Jenna R. Brown, Dennis R. Livesay

**Affiliations:** 1 Department of Biological Sciences, University of North Carolina at Charlotte, Charlotte, NC, 28262, United States of America; 2 Department of Bioinformatics and Genomics, University of North Carolina at Charlotte, Charlotte, NC, 28262, United States of America; UMR-S665, INSERM, Université Paris Diderot, INTS, FRANCE

## Abstract

β-lactamases are bacterial enzymes that confer resistance to β-lactam antibiotics, such as penicillins and cephalosporins. There are four classes of β-lactamase enzymes, each with characteristic sequence and structure properties. Enzymes from class A are the most common and have been well characterized across the family; however, less is known about how physicochemical properties vary across the C and D families. In this report, we compare the dynamical properties of four AmpC (class C) β-lactamases using our distance constraint model (DCM). The DCM reliably predicts thermodynamic and mechanical properties in an integrated way. As a consequence, quantitative stability/flexibility relationships (QSFR) can be determined and compared across the whole family. The DCM calculates a large number of QSFR metrics. Perhaps the most useful is the flexibility index (FI), which quantifies flexibility along the enzyme backbone. As typically observed in other systems, FI is well conserved across the four AmpC enzymes. Cooperativity correlation (CC), which quantifies intramolecular couplings within structure, is rarely conserved across protein families; however, it is in AmpC. In particular, the bulk of each structure is composed of a large rigid cluster, punctuated by three flexibly correlated regions located at the active site. These regions include several catalytic residues and the Ω-loop. This evolutionary conservation combined with active their site location strongly suggests that these coupled dynamical modes are important for proper functioning of the enzyme.

## Introduction

Antibiotic resistance continues to outpace our ability to produce new antibiotic drugs [1], leading to substantive fears about our future ability to combat bacterial infections that are currently relatively benign. Central to this growing global health concern is the bacterial enzyme β-lactamase (BL), which is produced by some bacteria [2]. BL confers resistance to penicillin and related antibiotics by hydrolyzing their conserved β-lactam moiety, thus destroying antibiotic activity [3]. The BL enzyme superfamily is broad and is characterized by varying degrees of antibiotic resistance activity. In fact, some BL enzymes confer resistance to cephalosporins, carbapenems, and monobactams [4, 5]. In particular, carbapenem-resistant enterobacteriaceae (CRE) that have KPC or NDM-1 carbapenamase genes represent a particularly dire and immediate biomedical concern [6]. The antibiotic spectrum of many BL enzymes has changed through active site mutations, most notably in the TEM [7], SHV [8], and GES [9] enzymes. The emergence of extended spectrum BL enzymes highlights the critical importance of understanding how physicochemical properties evolve across the BL superfamily [10, 11].

Comparisons of protein sequences and structures sharing function are well-established bioinformatics paradigms, leading to countless discoveries regarding sequence/structure/function relationships. Applied to antibiotic resistance enzymes, comparative studies group BL into four familial classes [12, 13]. Therein, class A, C and D enzymes share a common serine-based mechanism, whereas class B enzymes (sometimes called metallo-β-lactamases) rely on a metal ion mediated hydrolysis. There are discernable differences within sequence and structure across the family, even in the three serine-based classes. Sequence and structure clustering correlate with antibiotic resistance. For example, most class C enzymes correspond to cephalosporinases that are able to hydrolyze third generation cephalosporins [14, 15]. Consequently, elucidation of the differences and similarities between the various class C subfamilies is an important first step to fully understand the physical origins of the cephalosporinase activities across the family. Most of the biophysical characterizations into AmpC have focused either on elucidating its catalytic mechanism [16–20], elucidating stability/function trade-offs in active site residues [21], and—of course—identification of AmpC inhibitors (see [22] for a recent review). However, very little attention has been paid to AmpC’s dynamical properties.

While molecular dynamics simulations [17] and NMR [19] have been used to reveal mechanistic details, little is known about AmpC’s ambient equilibrium fluctuations. In response, we apply our computational distance constraint model (DCM) to four AmpC enzymes from the class C BL family. Specifically, we characterize AmpC enzymes from *E*. *coli*, *E*. *cloacae*, *C*. *freundii*, and *P*. *aeruginosa* for which there are available structures. As expected, backbone flexibility is well conserved across the family. However, in stark contrast to our previous results comparing structures across the class A [23] and class B1 [24] BL families—and several other protein families we have investigated—cooperativity correlation (CC) is also well conserved across all four AmpC enzymes. CC identifies all pairwise mechanical couplings within structure, meaning it can be considered to be a snapshot of allostery [25]. Consistent with experimental comparisons of allosteric couplings [26–30], our CC results are typically quite varied across a family [23, 31–34]. In fact, we have also demonstrated that CC can be significantly different across sets of protein mutants [35, 36]. As such, it is particularly noteworthy that CC is so well conserved in this system. The flexibly correlated regions define the perimeter of the active site, flanking both sides of the catalytic serine (Ser-64). Moreover, a third flexibly correlated region corresponds to the Ω-loop of the enzyme. These observations reinforce the notion that the mechanical couplings are functionally important, and suggest several new avenues of investigation in the role of dynamics in AmpC function and potential inhibition.

## Methods

### The Distance Constraint Model

The details of the DCM have been described many times previously [37–39], so only a cursory overview is provided here. The DCM is an all-atom statistical mechanical model that explicitly accounts for nonadditivity within free energy components [40] by directly accounting for enthalpy-entropy compensation [41, 42]. Starting from a native protein structure, we generate a network rigidity topological framework (graph) where vertices correspond to atoms, and edges represent chemical interactions that fix the distance between adjoining vertices. Weak interactions that continuously break and reform at ambient temperatures are allowed to fluctuate, whereas covalent bonds are quenched. An ensemble of frameworks ranging from fully folded to unfolded is generated by perturbing away from the original graph, the size of which is astronomically large (2^1900^ in the case of AmpC). As such, a mean field treatment is applied to the model [43, 44]. Specifically, two order parameters are defined to specify the number of H-bonds (salt bridges are treated as a special case of H-bonds) and the number of natively packed torsion angles, which correspond to the two types of fluctuating interactions currently modeled.

The free energy of a macrostate, defined by the number of H-bonds and number of native torsions (*N*_*hb*_,*N*_*nat*_), is given by Eq 1:

$$G\left(N_{hb},N_{nat}\right)=U\left(N_{hb}\right)-N_{hb}u_{sol}+N_{nat}v_{nat}-RTS_{cnf}\left(N_{hb},N_{nat}|\delta_{nat}\right)-RTS_{mix}\left(N_{hb},N_{nat}\right)$$ (Eq. (1))

where *R* is the universal gas constant, *T* is temperature, and *U*(*N*_*hb*_) is the total intramolecular H-bond enthalpy, which is calculated by an empirical potential [45]. The variables {*u*_*sol*_,*v*_*nat*_,*δ*_*nat*_} are phenomenological parameters that must be determined on a case-by-case basis, which is typically done by fitting to experimental heat capacity, *C*_*p*_, curves. The parameter *u*_*sol*_ describes compensating H-bonds to solvent when intramolecular H-bonds break, and {*v*_*nat*_,*δ*_*nat*_} correspond to the enthalpy and entropy associated with forming a native torsion. Corresponding values for the disordered torsions have been fixed in prior works and do not need to be parameterized [43, 44]. The total enthalpy of each framework is evaluated by summing the enthalpic components associated with each fluctuating constraint in the network. To account for nonadditivity within free energy components, the conformational entropy, *S*_*cnf*_, is calculated by summing only the components that correspond to the set of independent degrees of freedom (DOF), which are identified by a fast network rigidity graph algorithm [46, 47]. That is, excess (redundant) constraints that are not needed to rigidify a local region pay no entropic price upon formation because all of the DOF have been removed [41, 42]. In this way, enthalpy/entropy compensation is accurately described in a computationally tractable way. The mixing entropy, *S*_*mix*_, accounts for the various ways in which the macrostate can be satisfied. The partition function is constructed over the macrostates defined by the two order parameters, from which all thermodynamic response functions can be evaluated through appropriate derivatives thereof.

### Structure Preparation and Model Parameterization

**Fig 1:**
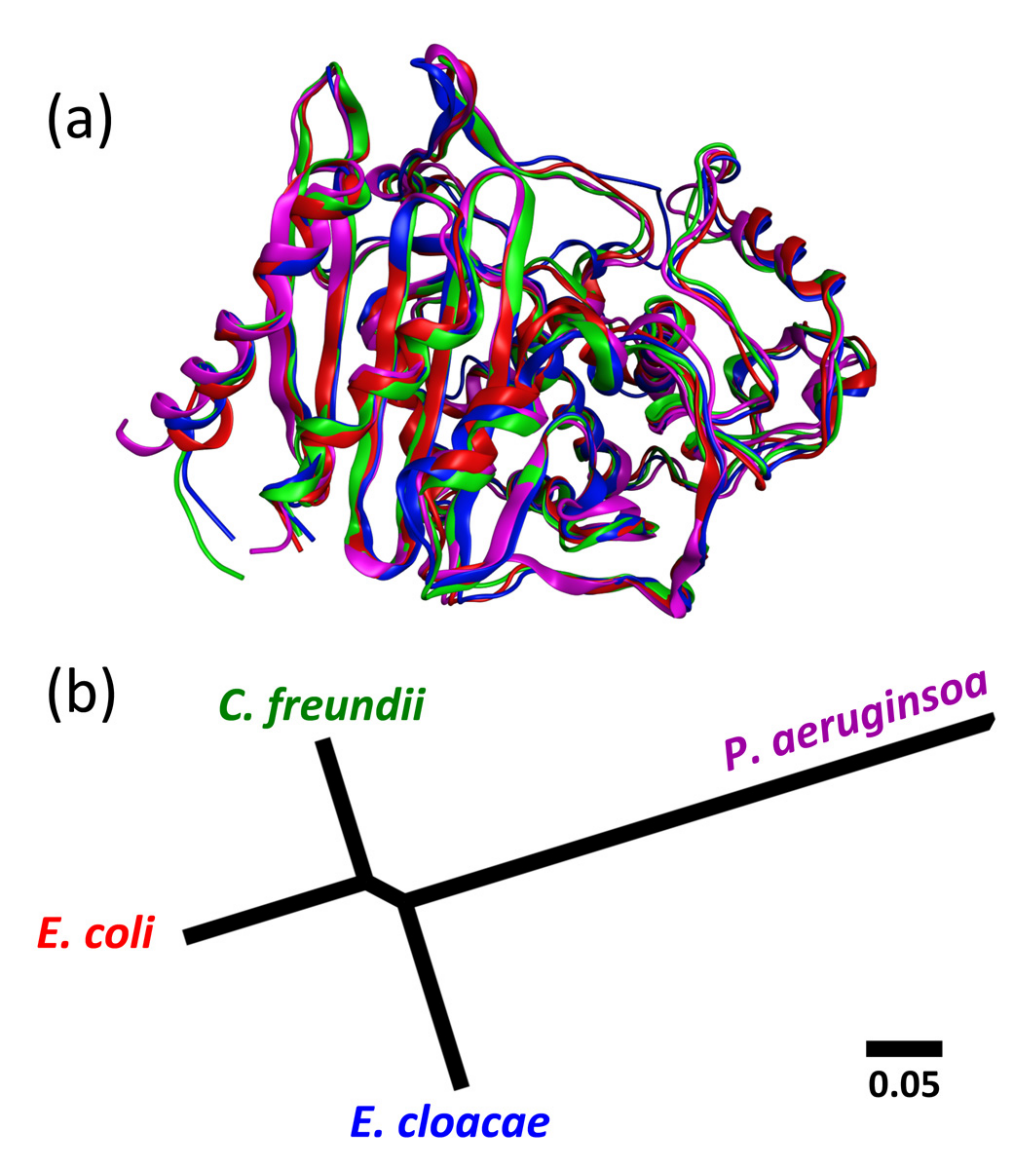
The four AmpC enzymes. (A) Superposition of the *E*. *coli* (red), *E*. *cloacae* (blue), *C*. *freundii* (green), and *P*. *aeruginosa* structures. (B) Neighbor-joining tree of the four considered enzymes.

AmpC structures are available for four organisms: *E*. *coli*, *E*. *cloacae*, C. *freundii*, and *P*. *aeruginosa*. More than 70 AmpC structures have been solved for *E*. *coli*, including more than 40 with an identical sequence. Conversely, only four *C*. *freundii* structures total are available. We parameterize the DCM for one structure from each of the four organisms (*E*. *coli* = 3GTC [48], *E*. *cloacae* = 1GA0 [49], *C*. *freundii* = 1FR6 [50], and *P*. *aeruginosa* = 2WZX [51]). The identified parameters are then applied to the other structures with the same sequence to evaluate prediction robustness. Fig 1A highlights the structural similarity within these four representative structures, and Fig 1B provides the Clustal Neighbor Joining tree [52] to reveal their sequence relationships. These results are listed in S1 Table. Before parameterization, hydrogen atoms are added and the structures are minimized in MOE using the Amber99 force field [53] and a distance dependent dielectric. Next, the H++ server is used to determine the appropriate ionization state at pH 7.0 based on calculated p*K*_*a*_ values.

Though AmpC experimental *C*_*p*_ data is unavailable for to fit the model too, the melting point, *T*_*m*_, and van't Hoff enthalpy of unfolding, Δ*H*_*unf*_, are available for the *E*. *coli* ortholog [54]. As such, we employ a grid search in parameter space over typical value ranges and select *E*. *coli* parameters that best correspond to the experimental data while maintaining typical shapes in the *C*_*p*_ curves and free energy landscapes (FELs). In the absence of experimental data for the *E*. *cloacae*, *C*. *freundii*, and *P*. *aeruginosa* enzymes, we again target the *E*. *coli* data, but emphasize the shapes of the *C*_*p*_ curves and FELs over *T*_*m*_ and Δ*H*_*unf*_ values.

### QSFR Metrics

After parameterization of the model and calculation of the thermodynamic response functions, the Boltzmann weights can be used to appropriately average a large number of mechanical properties over each macrostate. As such, the collective DCM output is referred to as Quantitative Stability/Flexibility Relationships (QSFR) based upon the way that thermodynamics and mechanics are fully integrated within the model [32]. It is typically more useful to collapse the two order parameters into a single global flexibility order parameter, θ, which quantifies the average number of independent degrees of freedom per residue. After identifying the native basins from the FELs expressed in terms of θ, many different mechanical properties are calculated and appropriately averaged. The two most useful quantities are the flexibility index (FI) and cooperativity correlation (CC). The FI quantifies backbone flexibility, where positive values quantify the number of excess DOF and negative values count the number of redundant constraints. Both are normalized so that they range between ±1. The FI is zero for isostatically rigid regions that have neither excess DOF nor redundant constraints; meaning removal of even a single constraint will cause the region to become flexible. CC is a higher order description of flexibility, where all pairwise mechanical couplings are evaluated. All residue pairs are considered, and residue pairs that are co-rigid or flexibly correlated are quantified.

## Results

### AmpC Comparisons

The AmpC enzymes are quite similar, with percent sequence identities across the *E*. *coli*, *E*. *cloacae*, *C*. *freundii*, and *P*. *aeruginosa* enzymes enzymes ranging from 42.5 to 77.4%. Similarly, they structurally superimpose with pairwise α-carbon RMSD values (calculated by MOE) of the four original structures ranging from 1.17 to 1.77 Å. While slight structural differences can be seen in the superposition (Fig 1A), particularly along loops, the overall backbone structure is well conserved. In fact, the differences across the four orthologs are not appreciably different from what is observed across the sets of structures with the same sequence (cf. S2 Table).

### Thermodynamic Properties

**Fig 2:**
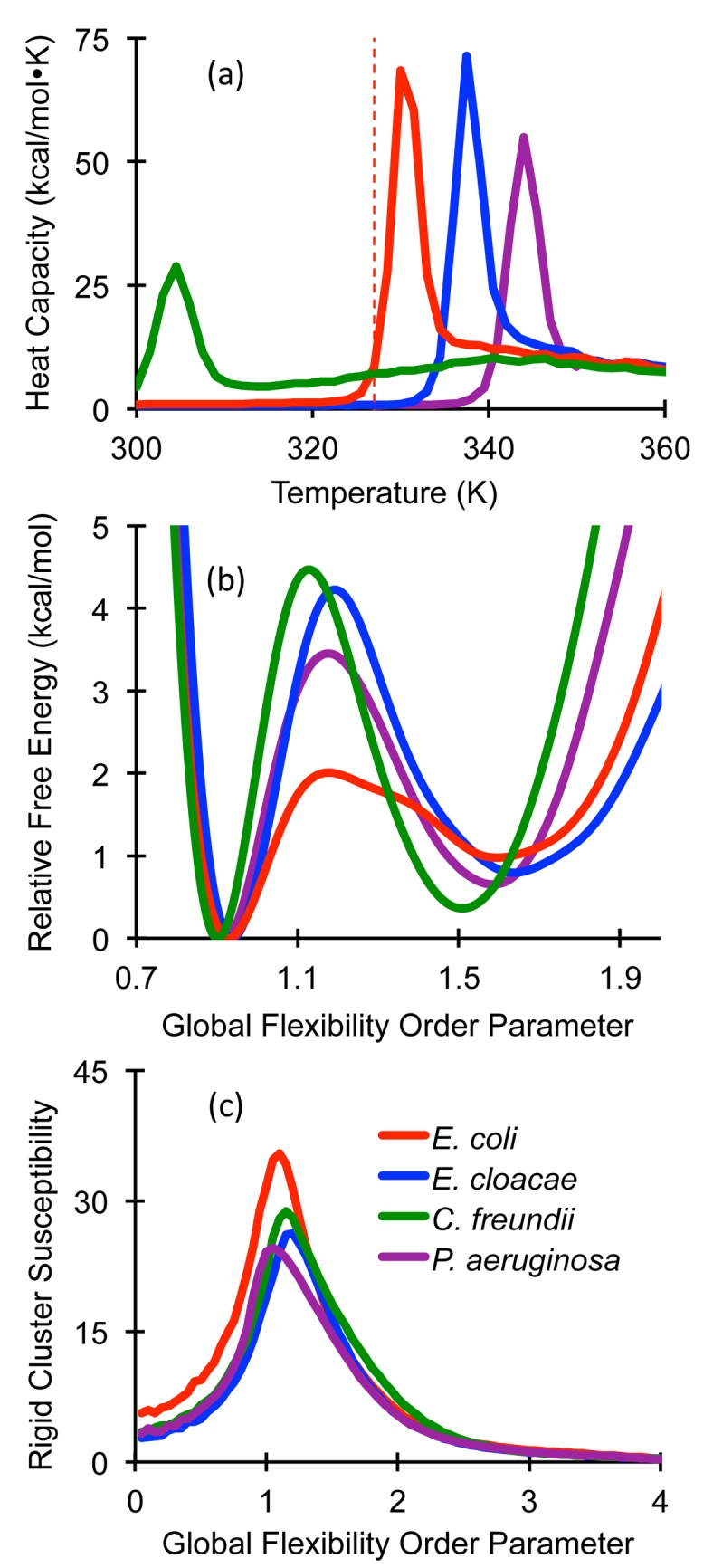
Physical descriptions of the original four AmpC structures. Thermodynamic descriptions of the unfolding transition are characterized by (A) heat capacity and (B) the free energy landscapes. The vertical dashed line indicates the experimental *T*_*m*_ value of the *E*. *coli* enzyme. The experimental Δ*H*_*unf*_ value is 182 kcal/mol, whereas the calculated value is 212 kcal/mol. (C) Rigid cluster susceptibility curves describe the mechanical transition for a structure predominantly composed of one large rigid cluster to many disjoint and floppy tiny clusters.

The model parameter values for the four original structures are provided in S3 Table, all of which are within typical ranges. Not surprising owing to their structural similarity, the parameter sets are remarkably similar. In fact, we often observe greater variance in the parameters for alternate structures of the exact same sequence. The predicted *C*_*p*_ curves are provided in Fig 2A. The *T*_*m*_ of the *E*. *coli* structure is satisfactorily similar to its experimental value, corresponding to less than 1% error on the Kevin scale. The model predicts the *C*. *freundii* enzyme to have a lowered *T*_*m*_, whereas the *E*. *cloacae and P*. *aeruginosa* enzymes are predicted to be slightly more stable. The FELs are provided in Fig 2B, which highlight the two-state nature of each folding transition. Notably, the θ _*nat*_ values, corresponding to the native basin free energy minima, are very well conserved.

Applying the above parameters to all structures that share the sequence with the original four, it is observed that the thermodynamic quantities can be sensitive to structure input. The average values and percent variance for the various thermodynamic quantities are provided in the supplemental material (S2 Table). The observed variability is in line with expectations as prior works have established that thermodynamic predictions can be sensitive to parameterization and structure. The percent variance within *T*_*m*_ and θ_*nat*_ values are always less than 15%, whereas the percent variance within the *C*_*p*_ peak heights is typically about 50%. The percent variance of the Δ*H* values for the *E*. *coli* and *E*. *cloacae* are in the middle of these two extremes, ranging from 20–25%, whereas the variation within the *C*. *freundii* and *P*. *aeruginosa* is about 70%.

### Rigidity Transition

Juxtaposed to the thermodynamic descriptions, mechanical descriptions of the unfolding transition are presented in Fig 2C. Herein, fluctuations within the rigid cluster sizes are indicated by the rigid cluster susceptibility (RCS) curves where the peak at θ_*rp*_ indicates the structure is transitioning from a predominately rigid structure to a flexible unfolded chain. The percent variation within the θ_*rp*_ values over all representative structures is always slightly less than 6%, which is similar to the variation within the θ_*nat*_ values. In all cases, θ_*rp*_ values greater than θ_*nat*_, indicating that the native structure is largely rigid since the mechanical transition has not yet occurred at the most probable value of θ.

### Backbone Flexibility and Cooperativity Correlation

**Fig 3:**
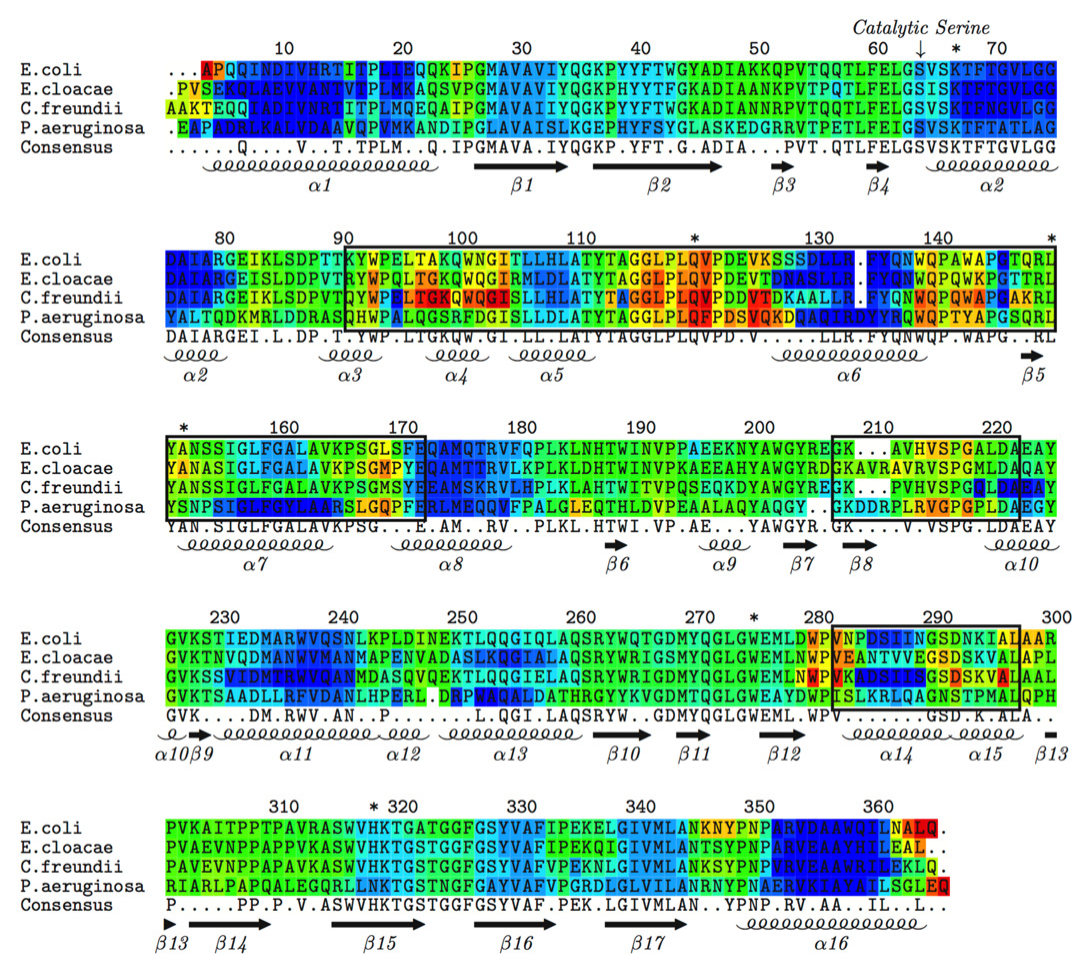
Multiple sequence alignment of the four AmpC enzymes color-coded by flexibility index. Red indicates positive values, corresponding to flexible regions, whereas blue indicates negative values, corresponding to rigid regions. Green indicates the flexibility index equals zero, corresponding to isostatically rigid regions. The catalytic Ser-64 is indicated by the arrow, whereas asterisks indicate the other active site residues. Boxes indicate the three flexibly correlated regions.

**Fig 4:**
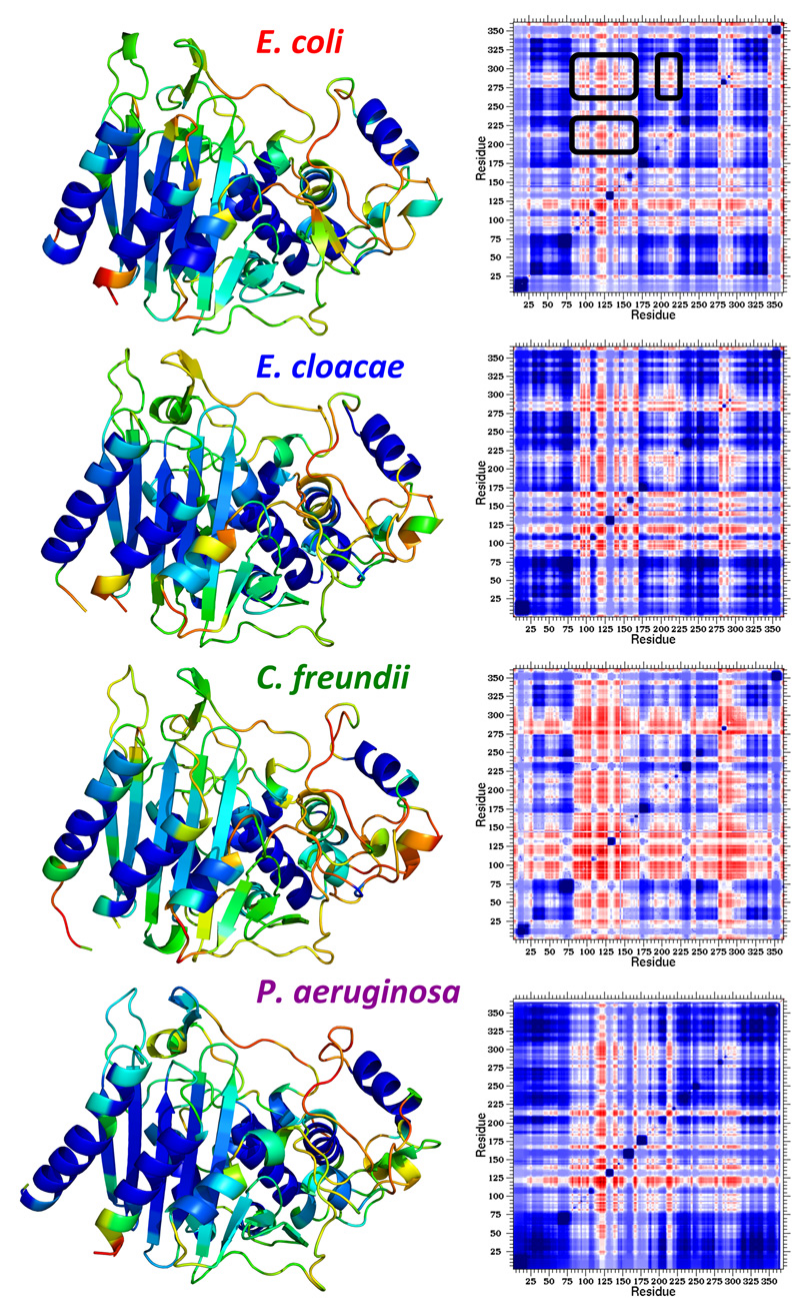
QSFR descriptions of the four AmpC enzymes. (Left) The structures of the four AmpC structures are color-coded in the same way as Fig 3. (Right) Cooperativity correlation plots for each of the four AmpC enzymes are shown, which are calculated as pixel-by-pixel averages across the sets of representative structures. Residue pairs that are co-rigid are colored blue; residues pairs that are flexibly correlated are colored red; and white indicates no mechanical coupling therein. The off-diagonal couplings indicating flexibility correlation is indicated by the black bands in the *E*. *coli* cooperativity correlation plot.

Mechanical properties are calculated by averaging over a sub-ensemble that corresponds to the native basin, which we do for each representative structure. Within each organism, the mechanical properties are then averaged to indicate the most probable descriptions. For example, in Fig 3 the color-coding within the multiple sequence alignment indicates the average FI values for each organism (blue indicates rigid, whereas red corresponds to flexible regions). Note, the *E*. *coli* AmpC values are averaged over 41 structures, whereas *E*. *cloacae*, *C*. *freundii*, and *P*. *aeruginosa* are averaged over five, four, and seven structures, respectively. In the cases of the *E*. *coli*, *E*. *cloacae*, and *P*. *aeruginosa*, the structures are taken from different PDB files. However, there is only two *C*. *freundii* structures with the same sequence, so we average over both the A and B chains in this case to generate more conformational diversity. Despite coming from the same structure, there is actually more conformational variability within the set of *C*. *freundii* structures than there is in the *E*. *coli* and *P*. *aeruginosa* structures (cf. S2 Table). In all four cases, secondary structures tend to be rigid. In fact, the most rigid regions correspond to α-helices, whereas β-strands are also rigid, albeit slightly less so. Intervening loops range from isostatically rigid (green) to flexible. The structures in Fig 4 are color-coded by FI using the same coloring scheme, further revealing the conserved backbone flexibility patterns. While the bulk of the native structure is rigid, the active site loops are among the most flexible portions of the enzyme. The average FI values ± 1 standard deviation for each of the four organisms are provided in the supplemental material (S1 Fig) to reveal the variation across the set of representative structures, highlighting the strong conservation therein.

**Fig 5:**
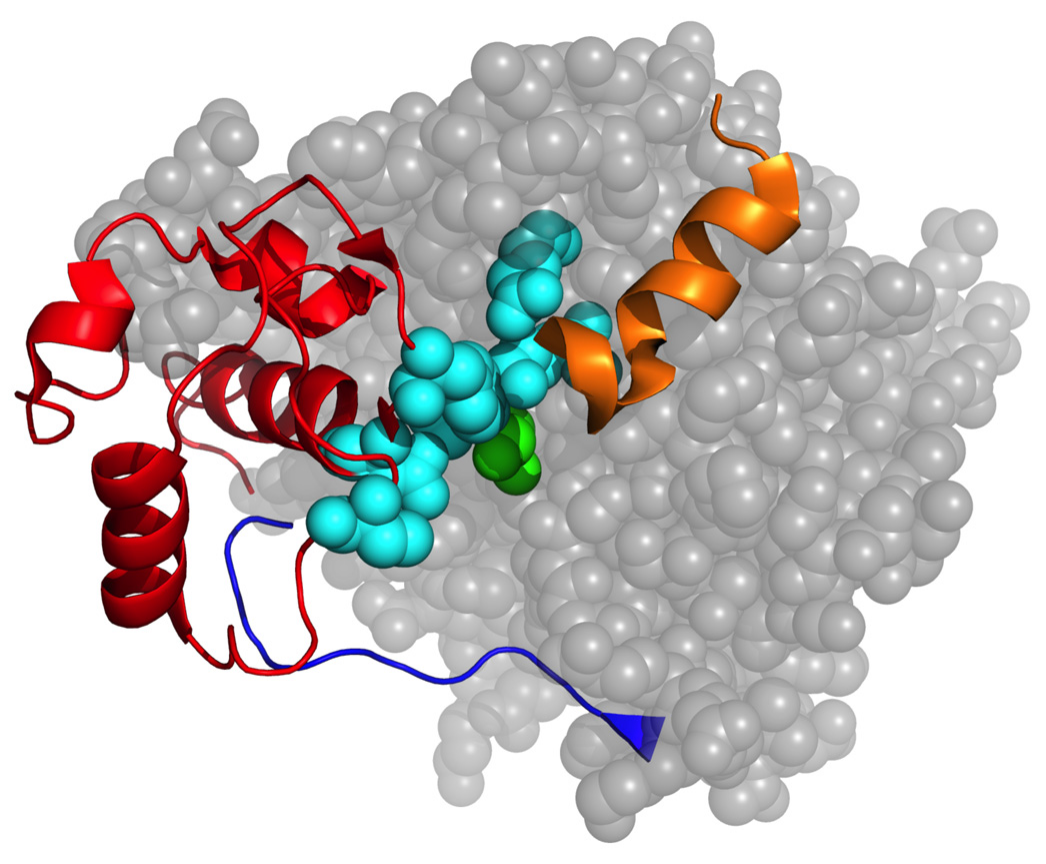
The conserved flexibly correlated active site regions. The structure of the *E*. *cloacae* AmpC enzyme is shown to highlight the consensus cooperativity correlation features. The grey residues shown in spacefill correspond to the large rigid cluster that makes up most of the enzyme; whereas the three active site regions are shown as cartoons (red = α-domain, blue = Ω-loop, and orange = α14 and α15 from the α/β-domain). The catalytic Ser-64 is colored green, whereas other active site residues are colored cyan.

Up to this point, the variations within thermodynamic and mechanical properties observed have been consistent with our prior results on alternate systems, including the class A and class B1 BLs. Typically, CC is quite variable across a family due to differences within the underlying H-bond networks. This is not the case with AmpC (cf. Fig 4). While quantitative pixel-to-pixel differences do occur, all four CC plots are characterized by a large rigid cluster (indicated in blue) punctuated by three bands of flexibility correlation. Actually, there is a bit more flexibility correlation within the *C*. *freundii* enzyme, but we only focus on the regions that are conserved across all four enzymes. The black boxes in Fig 3 bound the three conserved flexibly correlated regions, and are structurally highlighted in Fig 5. Using *E*. *cloacae* (1GA0), the regions include Arg-91 to Glu-171, Gly-206 to Ala-221, and Val-281 to Leu-296. The first region, which is the largest, overlaps much of the α-helical domain and includes several active site residues. In particular, Tyr-150, which has been shown to electrostatically stabilize the tetrahedral intermediate along the reaction coordinate [55], is within this region. The third region—in terms of sequence—corresponds to helices α14, α15, and the intervening loop, which are part of the α/β domain and contact the active site on the opposite side of the first. The last flexibly correlated band corresponds to most of the Ω-loop. The Ω-loop extends from the active site, and has been demonstrated to be critical to function [56]. Flexibility correlation between these regions is observed in all four enzymes, which is very unique. As stated, CC plots typically vary in both their patterns and the scale of the couplings. This atypical conservation within AmpC and their active site locations are strongly suggestive that these couplings are likely functionally important.

### QSFR Robustness and H-Bond Network Energy

**Fig 6:**
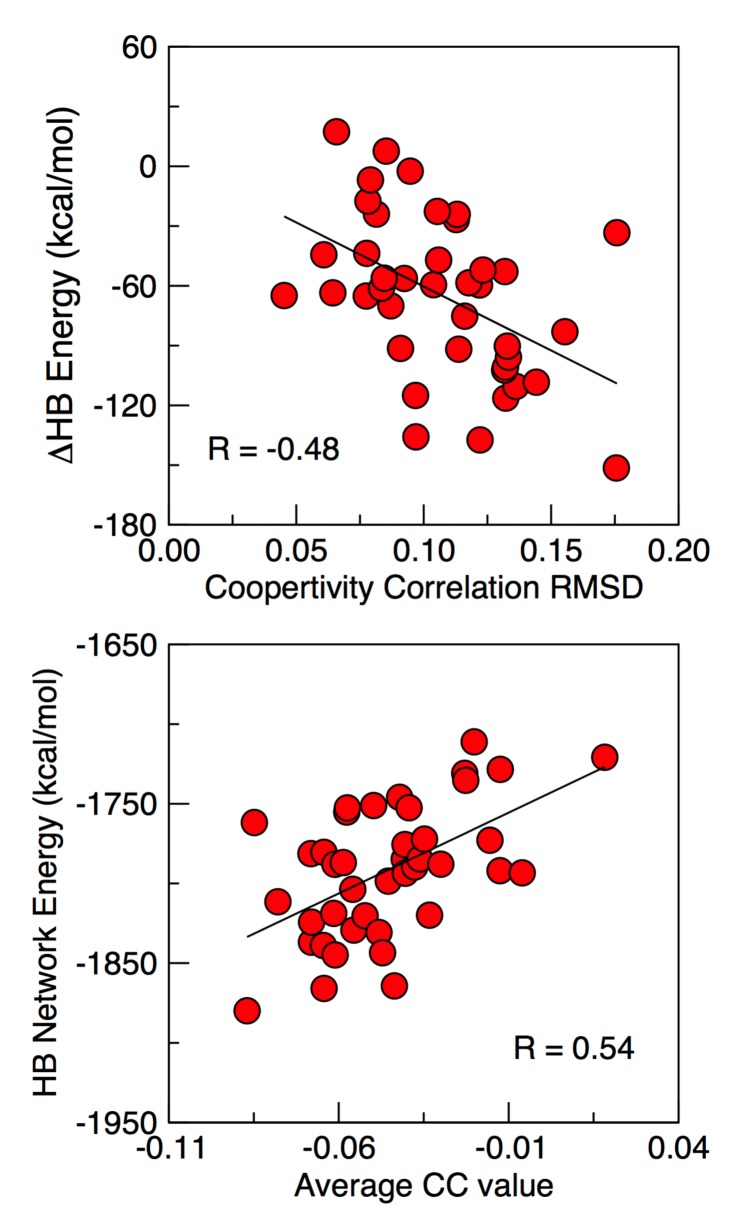
Relating cooperativity correlation variations to fluctuations within the hydrogen bond network. (Top) Scatter plot of the differences within the total H-bond network strength of the *E*. *coli* representative structures are plotted against cooperativity correlation similarity. In both cases, the original 3GTC structures are compared to each of the other representative structures. Cooperativity correlation similarity is calculated as the root mean square deviation (RMSD) across all pixels. (Bottom) The total H-bond network strength is plotted against the average CC value; large numbers indicate the CC plot is more red-shifted.

The variability within structural and QSFR properties across each set of representative structures is presented in S2 Table. Interestingly, the variances within the θ_*nat*_ and θ_*rp*_ values are actually greatest in the case of *E*. *coli* despite the fact that it has eight times more structures. A similar trend can be observed in the CC plots. All of the *E*. *cloacae* and *P*. *aeruginosa* CC plots are visually similar, whereas there are a few obvious outliers in the case of *E*. *coli*. In fact, a small number of the *E*. *coli* plots can be quite red or blue-shifted. The source of the shifts is based on differences within the H-bond network energies. Fig 6 plots the difference in the total H-bond energy for each structure (with respect to 3GTC) versus the differences within CC plots, revealing a significant linear correlation. That is, the stronger the H-bond network, the more co-rigid the structure is predicted to be, whereas weaker H-bond networks lead to more correlated flexibility. The CC plots of the three *E*. structures with the strongest H-bond energies to the three CC plots with the weakest are compared in the supplemental material (S2 Fig); the CC plot of 3GTC and the average plot are also provided as reference points.

Not surprisingly, the total H-bond energy is also linearly related to the values of θ_*nat*_. That is, structures with stronger H-bond energies correspond to fewer independent DOF per residue in their native structure (cf. S3 Fig). Interestingly, when CC plots are compared using Pearson correlations instead of RMSD, the relationship between θ_*nat*_ and H-bond network energy is drastically reduced. In many cases, strengthening/weakening of the H-bond network can lead to significant blue- or red-shifting of the CC plots, even though the overall features are mostly conserved. Conversely, there are other examples where the overall color and θ_*nat*_ are conserved; however, a few extra features in the CC plot appreciably reduce the correlation (i.e., flexibility correlation along the N- and C- termini). The box plots of the actual CC values for every pixel in eight exemplar plots are provided in S4 Fig, which quantifies the color shifting.

### Parameter Sensitivity

A natural question is whether or not the discussed QSFR properties are biased by parameterization. Our prior works have demonstrated that this is not the case so long as the parameters are within typically expected ranges [32, 57]. Nevertheless, this possibility should always be confirmed. The easiest way to do so is to swap the parameters from one enzyme and apply them to the others, which maintains the correlations that are present within the parameter set. That is, simply changing one parameter while holding the other two fixed is typically more disruptive than allowing two or three to adjust together because there are compensations within the changes. The robustness within the considered QSFR properties is highlighted in S5 Fig, which compares the original FI and CC values to the two “swapped” parameter sets for *E*. *coli* (similar conservation is observed in the other two cases).

Correlation coefficients comparing each of the four original structures to itself are provided in Table 1, but using one of the other parameter sets. In all cases, the correlations for FI are greater than 0.998, and the correlations for CC are greater than 0.995, meaning the slight parameter differences across the AmpC enzymes have virtually no effect on the mechanical properties. Note that the average correlation comparing FI and CC across the sets of representative structures is 0.720 and 0.725, respectively (the percent variance is 10.3 and 7.8%). That is, conformational changes while holding model parameters fixed are significantly more disruptive than swapping parameters on a given structure, meaning the employed parameter-swapping test confirms that the relative differences in QSFR properties are not simply due to parameterization. Note that the parameter differences do have a small effect on the thermodynamic descriptions, which are more sensitive than the mechanical properties. Thermodynamics controls the relative probability of the folded to the unfolded basins; however, the θ values of the basins do not shift very much, which is why there is sensitivity in the thermodynamics, but not the mechanics.

**Table 1:** QSFR sensitivity to model parameters.

Cooperativity Correlation				
	*E*. *coli*	*E*. *cloacae*	*C*. *freundii*	*P*. *aeruginosa*
*E*. *coli*	—	0.9918	0.9959	0.9980
*E*. *cloacae*	0.9974	—	0.9998	0.9983
*C*. *freundii*	0.9970	0.9952	—	0.9980
*P*. *aeruginosa*	0.9993	0.9984	0.9955	—
Flexibility Index				
	*E*. *coli*	*E*. *cloacae*	*C*. *freundii*	*P*. *aeruginosa*
*E*. *coli*	—	0.9995	0.9990	0.9988
*E*. *cloacae*	0.9983	—	1.0000	0.9995
*C*. *freundii*	0.9995	0.9983	—	0.9997
*P*. *aeruginosa*	0.9995	0.9996	0.9987	—

Parameter sets along the horizontal are applied to structures on the vertical, and then the QSFR properties are compared using the Pearson correlation.

## Discussion

In prior works we have characterized QSFR properties across twelve class A [23] and five class B1 [24] enzymes using the DCM. In both cases, backbone flexibility is largely conserved, whereas the pairwise intramolecular couplings described by CC are quite variable. Differences within the global flexibility properties of the class A enzymes do parallel the phylogeny of the family; however, they do not correlate with antibiotic specificities. This result is particularly interesting because it indicates that extended-spectrum activities are not constrained by global properties. Rather, cephalosporinase and carbapenamase activities can evolve from a very wide selection of global properties. An equally interesting revelation from our comparative studies of the class B1 enzymes is that the active site β3/β4 and β11/α6 loops are flexibly correlated in all four structures. However, overall the CC plots are very different. For example, NDM-1 has no other appreciable flexibility correlation, whereas the VIM-4 and IMP-1 CC plots are both significantly red-shifted.

While the class A enzyme TEM-1 is known to be quite rigid [58], which our results showed, its active site Ω-loop is known to be quite mobile [59–61]. However, our results predict the Ω-loop across the whole family to be nearly isostatically rigid, which highlights the difference between mobility and flexibility. That is, the Ω-loop is mobile and dynamic, but it moves through space as a rigid body. The Ω-loops in the four AmpC structures are similarly isostatic, which suggests that the Ω-loop acts as a mechanical switch because it is nearly perfectly balanced between rigid and flexible. That is, it can easily transition from flexible to rigid, and vice versa, by very subtle fluctuations within the H-bond network. Even the hinges are mostly isostatic, meaning the Ω-loop can be rigidly locked in place or quite dynamic, which are functional requirements of a conformational switch.

The Ω-loop corresponds to one of three flexibly correlated regions revealed by the CC plots, meaning when it is flexible it is flexibly correlated to the other two regions. However, when it is rigid, it is not part of the large rigid cluster that makes up most of the structure. The largest of the three regions corresponds to the α-helical domain, and includes the active site Gln-120, Tyr-150, and Gln-152 residues. This point is noteworthy because, unlike Glu-166 of the class A enzymes (which is located on the Ω-loop) [62], AmpC is not thought to have a specific general base that activates the catalytic Ser-64. Rather, it has been suggested that several active site residues collectively activate the enzyme through a H-bond network [18] that includes the three residues within the flexibly correlated α-domain, plus other active site residues Lys-67, Glu-275, and Lys-318. This point is intriguing because—as demonstrated above—the H-bond network principally defines the QSFR properties due to its crosslinking nature (in contrast to the local nature of covalent bonds and the torsion interactions). The convergence of the active site electrostatic network and the conserved dynamical properties suggests that the flexibility correlation observed in all four AmpC enzymes is a mechanistic requirement. That is, it is possible that fluctuations within the active site conformations adjust the electrostatic microenvironments [63–65] such that active site can function as a charge relay system [18].

While compelling, the last point is only a hypothesis. Nevertheless, it is clear that—like the class A TEM-1 enzyme—the native structure of each AmpC is primarily composed of a single rigid cluster that spans both domains. Moreover, owing to their conservation and active site proximity, it is nearly certain that the flexibility correlations within the three active site regions are functionally important. A remaining open question is why does this atypical QSFR conservation occur in AmpC. It is possible that it is a functional requirement for all AmpC enzymes, or it could be do to the fact that the four considered AmpC enzymes are relatively closely related (they all occur in Proteobacteria). Recall, that while the QSFR properties in the class-A enzymes are overall variable, the properties are mostly conserved within evolutionary outgroups. Unfortunately this will only be resolved after more distantly related AmpC structures have been structurally characterized.

## Supporting Information

S1 Fig Backbone flexibility profile. Average flexibility index values are plotted versus sequence position, with ± one standard deviation shown to highlight the variance within backbone flexibility across the set of representative structures.
(TIF)

S2 Fig Cooperativity correlation variation. Examples of three most blue-shifted and three most red-shifted *E*. *coli* cooperativity correlation plots are shown. The original 3GTC and the average cooperativity correlation plots are also shown as reference points.
(TIF)

S3 FigScatter plots comparing differences within the *E*. *coli* representative structural properties to differences within cooperativity correlation.In all cases, the original 3GTC structure is compared each of the other representative structures. The y-axis quantities are: (Top) $\theta_{nat}^{trial}-\theta_{nat}^{3GTC}$, (Middle) ${\left[\theta_{nat}-\theta_{rp}\right]}^{trial}-{\left[\theta_{nat}-\theta_{rp}\right]}^{3GTC}$, and (Bottom) $U_{hb}^{trial}-U_{hb}^{3GTC}$. In the left column, cooperativity correlation is evaluated by the pixel-to-pixel root mean square deviation (RMSD), whereas the Pearson correlation coefficient is used in the right column.(TIF)

S4 Fig Cooperativity correlation distributions. The raw value distributions taken from the cooperativity correlation plots in S3 Fig are plotted as box plots. The labels of the three blue-shifted structures are colored blue; the labels of the red-shifted are colored red; and the original 3GTC structure and average plot are colored black.
(TIF)

S5 Fig Parameter sensitivity. The flexibility index and cooperativity correlation plots for the 3GTC (*E*. *coli*) structure using the original parameter values are, respectively, compared to the 1GA0 (*E*. *cloacae*) and 1F46 (*C*. *freundii*) parameter sets, which have the most extreme differences across the four parameter sets used. In the top row, the flexibility index is compared (black = original, red = 1GA0 parameters, and green = 1FR6 parameters). The next three rows show the three cooperativity correlation plots as indicated.
(TIF)

S1 Table List of representative structures characterized. Asterisks indicate structures used to parameterize the model. Unless noted otherwise, only A-chain structures are used from each crystal structure.
(DOCX)

S2 Table Variation within various physical properties across the representative structures. Presented quantities are averaged over the set of representative structures for each AmpC enzyme, and percent variation (standard deviation/average x 100) is used to quantify the variation therein. Top values correspond to averages, whereas, percent variance is provided in the bottom row when appropriate.
(DOCX)

S3 Table Model parameters. A brief description of the three phenomenological parameters and the employed values for each AmpC enzyme are provided.
(DOCX)
