# Supplementary figures and images for: Flexibility Correlation between Active Site Regions Is Conserved across Four AmpC β-Lactamase Enzymes
Source: PLoS One. 2015 May 27;10(5):e0125832. doi: 10.1371/journal.pone.0125832 (PMC4446314; doi:10.1371/journal.pone.0125832)

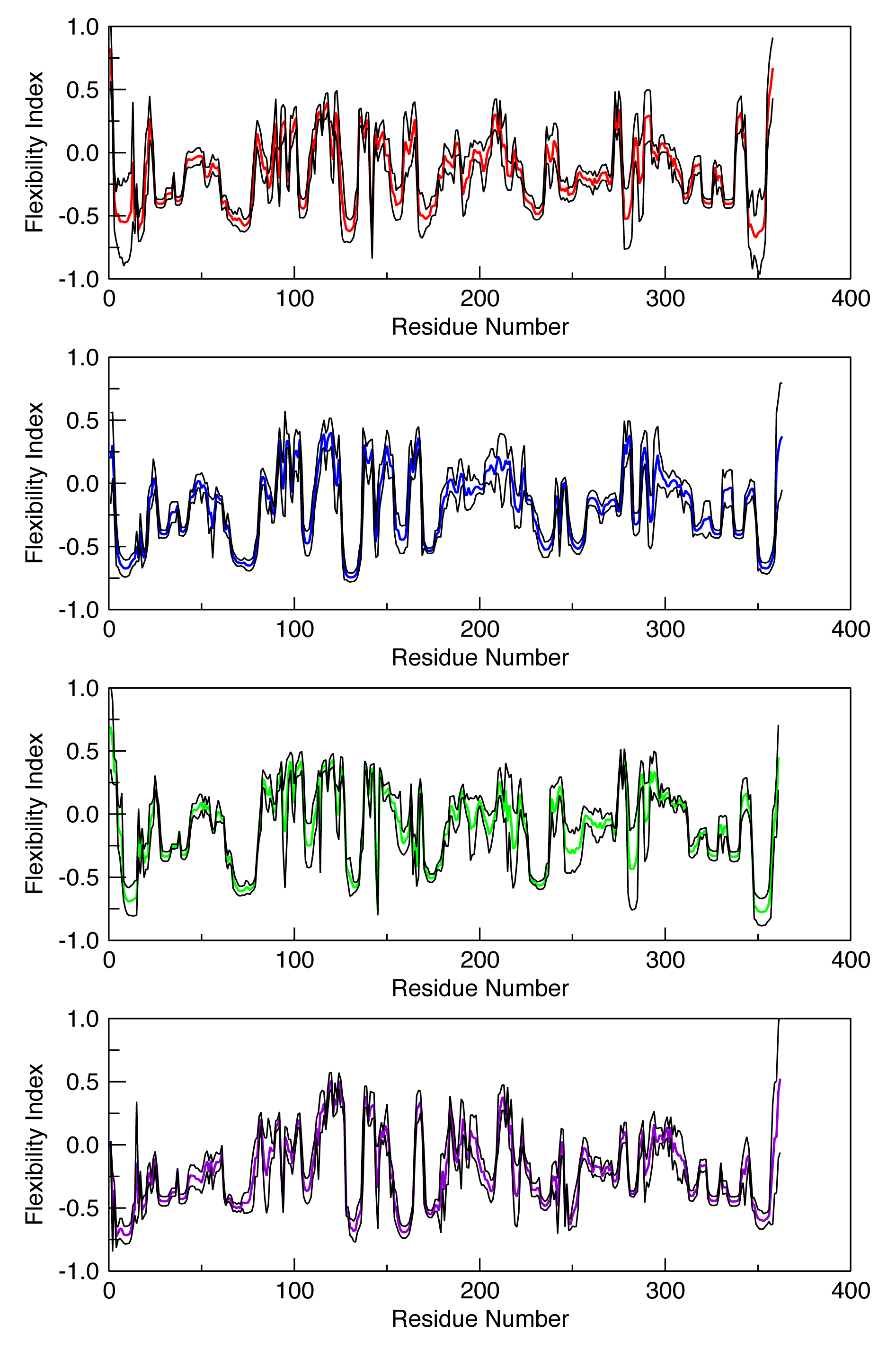

Supplement: S1 Fig — Average flexibility index values are plotted versus sequence position, with ± one standard deviation shown to highlight the variance within backbone flexibility across the set of representative structures. (TIF) [file pone.0125832.s001.tif]

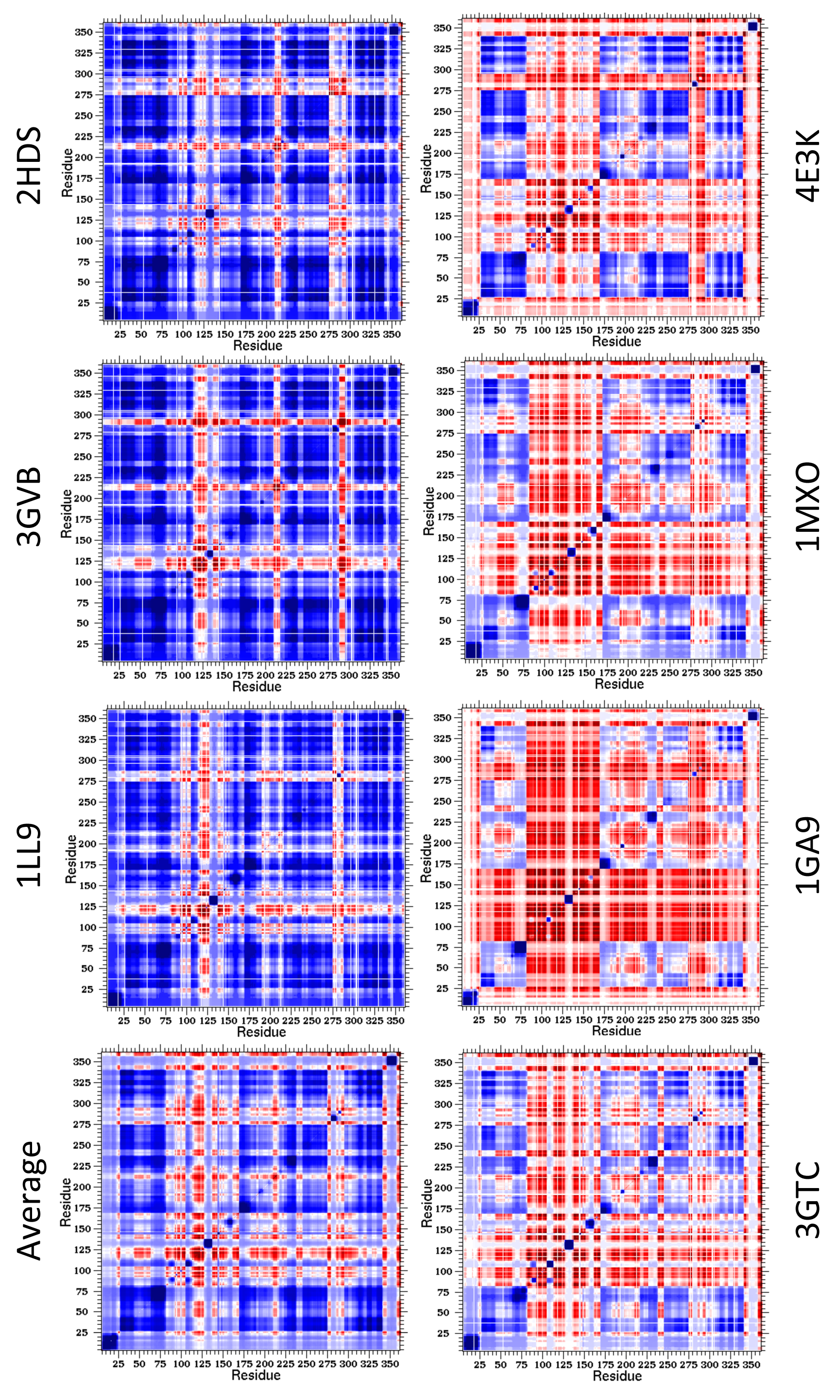

Supplement: S2 Fig — Examples of three most blue-shifted and three most red-shifted E. coli cooperativity correlation plots are shown. The original 3GTC and the average cooperativity correlation plots are also shown as reference points. (TIF) [file pone.0125832.s002.tif]

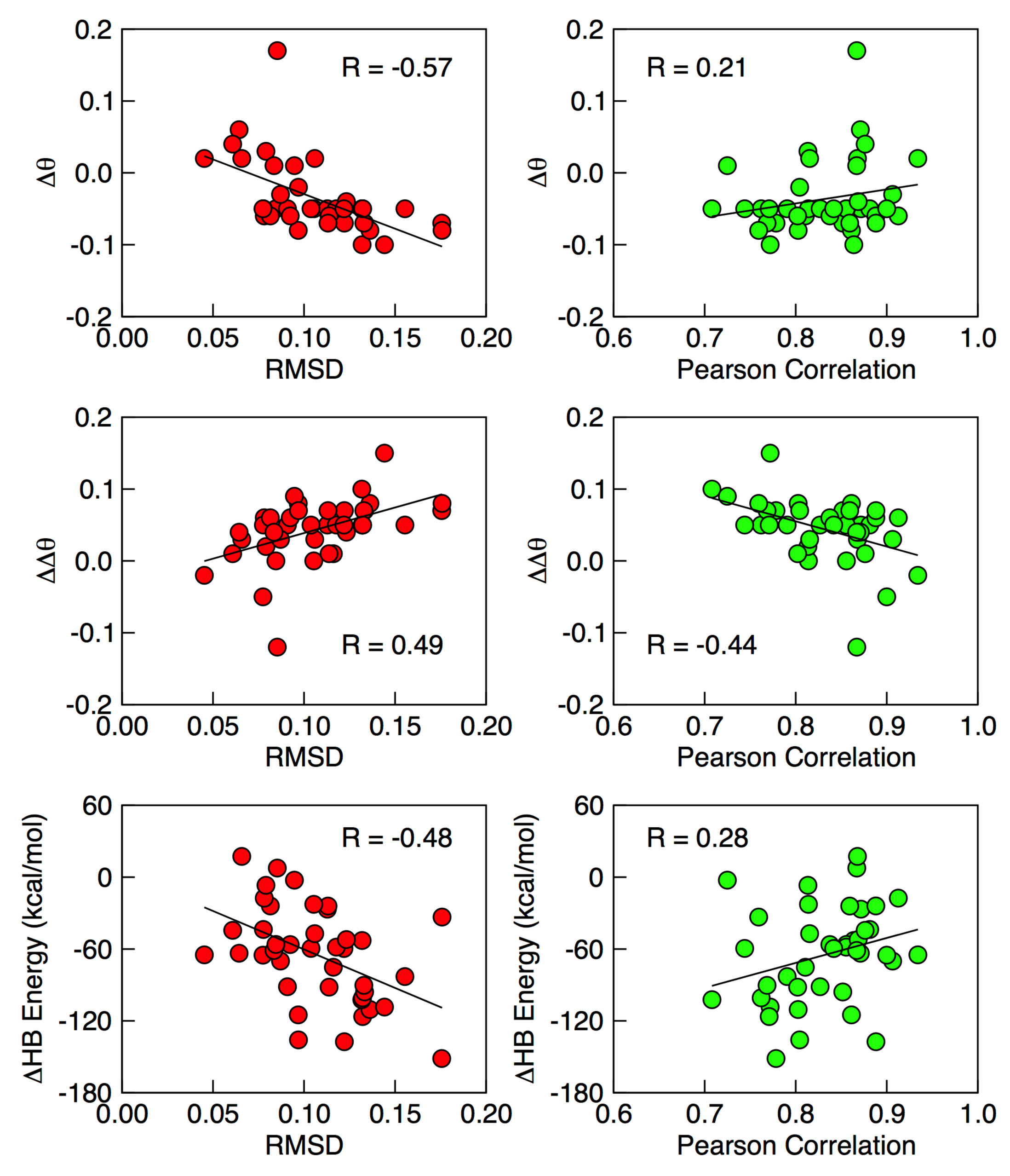

Supplement: S3 Fig — In all cases, the original 3GTC structure is compared each of the other representative structures. The y-axis quantities are: (Top) θnattrial−θnat3GTC, (Middle) [θnat−θrp]trial−[θnat−θrp]3GTC, and (Bottom) Uhbtrial−Uhb3GTC. In the left column, cooperativity correlation is evaluated by the pixel-to-pixel root mean square deviation (RMSD), whereas the Pearson correlation coefficient is used in the right column. (TIF) [file pone.0125832.s003.tif]

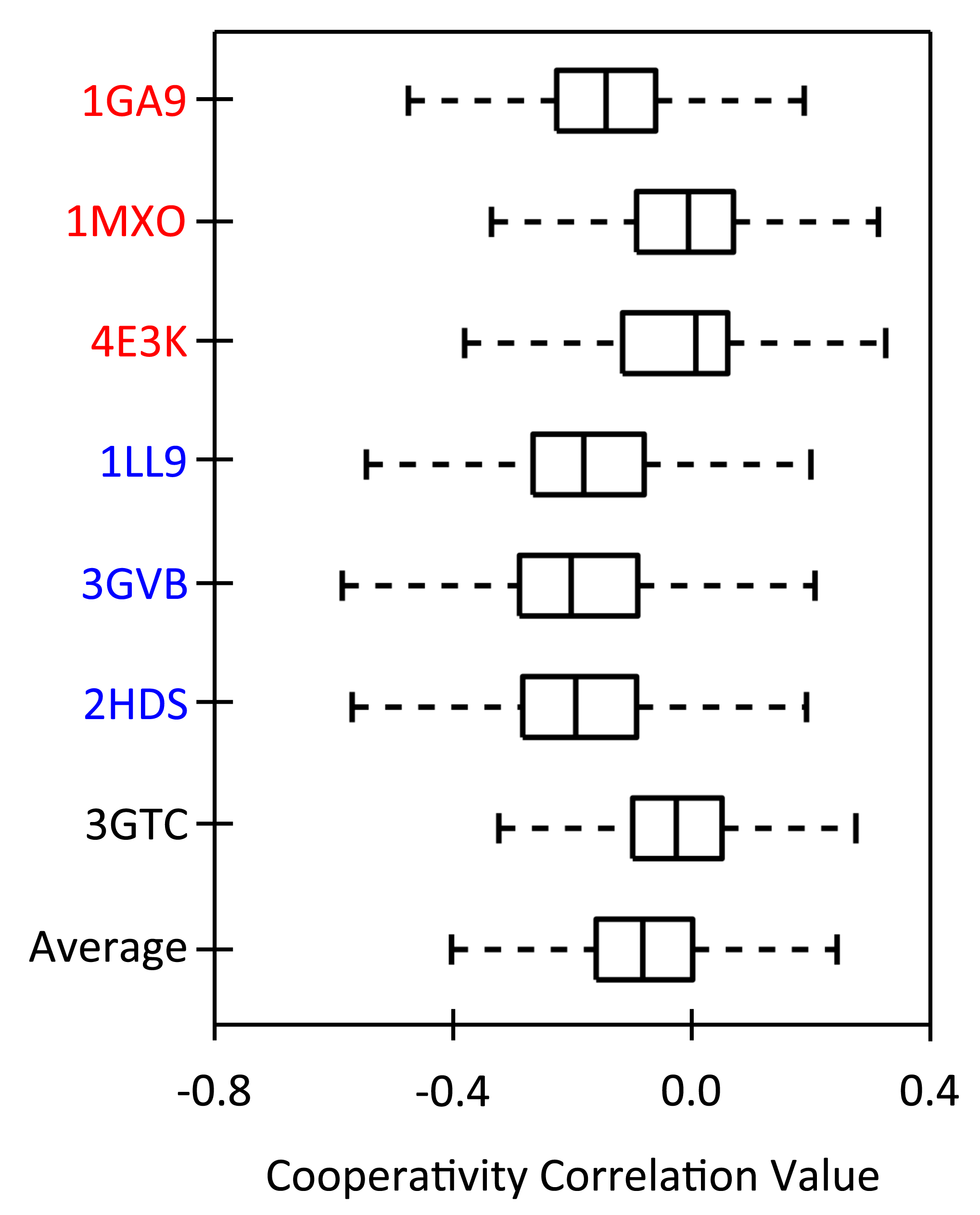

Supplement: S4 Fig — The raw value distributions taken from the cooperativity correlation plots in S3 Fig are plotted as box plots. The labels of the three blue-shifted structures are colored blue; the labels of the red-shifted are colored red; and the original 3GTC structure and average plot are colored black. (TIF) [file pone.0125832.s004.tif]

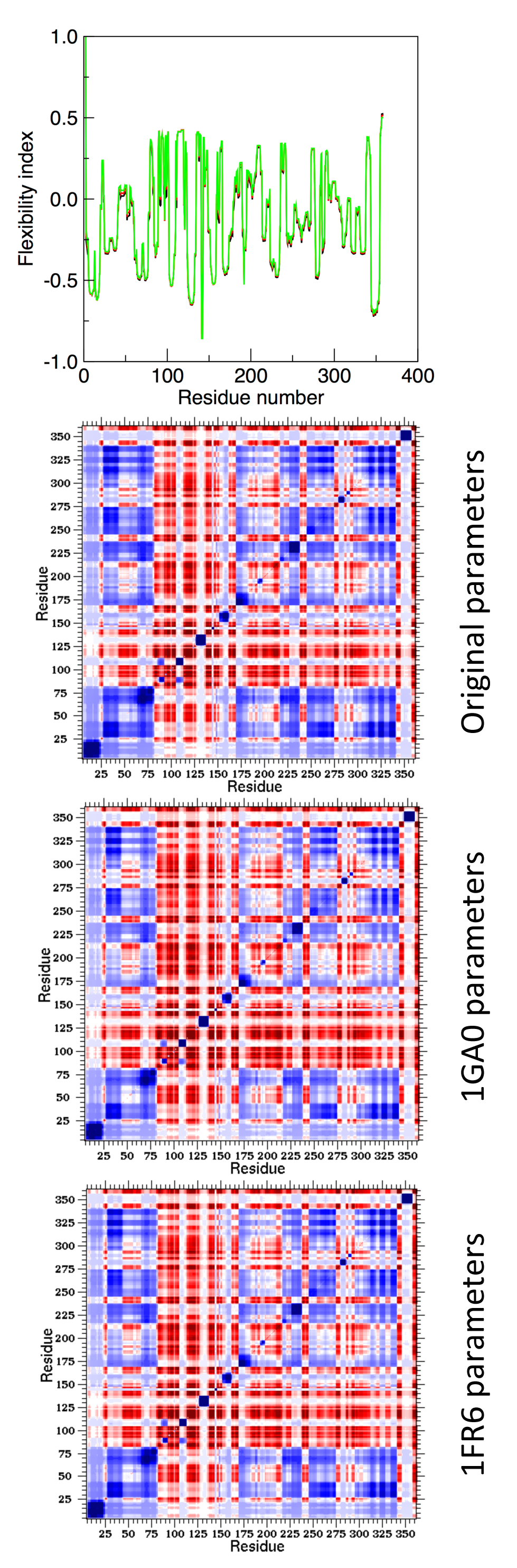

Supplement: S5 Fig — The flexibility index and cooperativity correlation plots for the 3GTC (E. coli) structure using the original parameter values are, respectively, compared to the 1GA0 (E. cloacae) and 1F46 (C. freundii) parameter sets, which have the most extreme differences across the four parameter sets used. In the top row, the flexibility index is compared (black = original, red = 1GA0 parameters, and green = 1FR6 parameters). The next three rows show the three cooperativity correlation plots as indicated. (TIF) [file pone.0125832.s005.tif]
