# Supplementary material for: Flexibility Correlation between Active Site Regions Is Conserved across Four AmpC β-Lactamase Enzymes
Source: PLoS One. 2015 May 27;10(5):e0125832. doi: 10.1371/journal.pone.0125832 (PMC4446314; doi:10.1371/journal.pone.0125832)
Supplement: S1 Table — Asterisks indicate structures used to parameterize the model. Unless noted otherwise, only A-chain structures are used from each crystal structure. (DOCX) [file pone.0125832.s006.docx]

Table S1. List of representative structures characterized

| *E. coli* | *E. cloacae* | *C. freundii* | *P. aeruginosa* |
| --- | --- | --- | --- |
| 3GTC* | 1GA0* | 1FR6:A* | 2WZX* |
| 1C3B | 1GCE | 1FR6:B | 2WZZ |
| 1FCO | 1ONH | 1FR1:A | 3S1Y |
| 1GA9 | 1Q2Q | 1FR1:B | 3S22 |
| 1IEL | 1RGZ |  | 4GZB |
| 1IEM |  |  | 4NK3 |
| 1KDS |  |  | 4OOY |
| 1KDW |  |  |  |
| 1KE0 |  |  |  |
| 1KE3 |  |  |  |
| 1KE4 |  |  |  |
| 1KVM |  |  |  |
| 1L2S |  |  |  |
| 1LL5 |  |  |  |
| 1LL9 |  |  |  |
| 1LLB |  |  |  |
| 1MXO |  |  |  |
| 1XGI |  |  |  |
| 1XGJ |  |  |  |
| 2HDQ |  |  |  |
| 2HDR |  |  |  |
| 2HDS |  |  |  |
| 2HDU |  |  |  |
| 2I72 |  |  |  |
| 2P9V |  |  |  |
| 2PU2 |  |  |  |
| 2R9W |  |  |  |
| 2R9X |  |  |  |
| 2RCX |  |  |  |
| 3BM6 |  |  |  |
| 3GR2 |  |  |  |
| 3GSG |  |  |  |
| 3GVB |  |  |  |
| 3O86 |  |  |  |
| 3O88 |  |  |  |
| 4E3I |  |  |  |
| 4E3J |  |  |  |
| 4E3K |  |  |  |
| 4E3L |  |  |  |
| 4E3M |  |  |  |
| 4E3O |  |  |  |
