# Supplementary material for: Flexibility Correlation between Active Site Regions Is Conserved across Four AmpC β-Lactamase Enzymes
Source: PLoS One. 2015 May 27;10(5):e0125832. doi: 10.1371/journal.pone.0125832 (PMC4446314; doi:10.1371/journal.pone.0125832)
Supplement: S2 Table — Presented quantities are averaged over the set of representative structures for each AmpC enzyme, and percent variation (standard deviation/average x 100) is used to quantify the variation therein. Top values correspond to averages, whereas, percent variance is provided in the bottom row when appropriate. (DOCX) [file pone.0125832.s007.docx]

Table S2. Variation within various physical properties across the representative structures

|  | *E. coli* | *E. cloacae* | *C. freundii* | *P. aeruginosa* |
| --- | --- | --- | --- | --- |
| Number of representative structures | 41 | 5 | 4 | 7 |
| Structural Properties | | | | |
| Pairwise structural RMSD (Å) | 0.88  17.3% | 1.16  18.3% | 1.03  25.0% | 0.84  18.4% |
| Crystal Structure Resolution (Å) | 1.5  12.4% | 1.9  14.6% | 2.3  15.7% | 1.5  17.5% |
| Crystal Structure R-value | 0.16  18.2% | 0.18  11.6% | 0.19  0.4% | 0.18  4.6% |
| DCM Calculated Properties | | | | |
| *T_m_* values (K) | 326.5  2.6% | 319.7  3.9% | 295.0  5.0% | 345.6  14.0% |
| *C_p_* peak height (kcal/mol•K) | 64.0  41.3% | 60.1  48.9% | 14.5  66.6% | 66.8  54.8% |
| Δ*H_unf_* values (kcal/mol) | 330.2  19.7% | 319.7  24.6% | 145.3  68.6% | 200.7  69.4% |
| θ*_nat_* | 0.89  5.7% | 0.91  3.0% | 1.03  4.4% | 0.85  4.2% |
| θ*_rp_* | 1.11  3.6% | 1.17  2.3% | 1.18  5.5% | 1.08  2.5% |
