# Supplementary material for: Flexibility Correlation between Active Site Regions Is Conserved across Four AmpC β-Lactamase Enzymes
Source: PLoS One. 2015 May 27;10(5):e0125832. doi: 10.1371/journal.pone.0125832 (PMC4446314; doi:10.1371/journal.pone.0125832)
Supplement: S3 Table — A brief description of the three phenomenological parameters and the employed values for each AmpC enzyme are provided. (DOCX) [file pone.0125832.s008.docx]

Table S3. Model parameters

| Parameter | Description and units | *E. coli*  3GTC | *E. cloacae*  1GA0 | *C. freundii*  1FR6:A | *P. aeruginosa*  2WZX |
| --- | --- | --- | --- | --- | --- |
| *u_sol_* | H-bond to solvent (kcal/mol) | -2.04 | -1.93 | -1.93 | -1.95 |
| *v_nat_* | Natively packed torsion enthalpy (kcal/mol) | -0.20 | -0.10 | -0.10 | -0.15 |
| *δ_nat_* | Natively packed torsion pure entropy (unitless) | 1.52 | 1.50 | 1.52 | 1.52 |
